# Explaining Perceived Priorities in Women with Breast Cancer: A Qualitative Study

**DOI:** 10.31557/APJCP.2019.20.11.3311

**Published:** 2019

**Authors:** Seyede Zahra Ghaemi, Zohreh Keshavarz, Sedigheh Tahmasebi, Majid Akrami, Seyed Taghi Heydari

**Affiliations:** 1 *Student Research Committee, *; 2 *Department of Midwifery and Reproductive Health, School of Nursing and Midwifery, Shahid Beheshti University of Medical Sciences, Tehran, *; 3 *Department of General Surgery, Lymphedema Research Center, *; 4 *Breast Diseases Research Cancer, Department of Surgery, *; 5 *Health Policy Research Center, School of Medicine, Shiraz University of Medical Sciences, Shiraz, Iran. *

**Keywords:** Perceived priority, support, breast neoplasms, breast cancer

## Abstract

**Objective:**

Cancer is a stressful event in life, and the dreadful impact and problems created for patients and families by cancer negatively affect their quality of life. Therefore, regarding the increasing number of cancer patients and the nature of this disease, the need to recognize and understand the priorities and problems of patients after the diagnosis of cancer is of high importance. This study was designed and implemented with the aim of identifying the perceived priorities of women with breast cancer.

**Methods:**

This study is a qualitative research of content analysis type. To collect data, purposeful sampling and deep semi-structured individual interviews were used. The subjects were women with breast cancer who visited the Breast Disease Research Center of Shahid Motahari Clinic in Shiraz, and the data were saturated after 15 interviews. The four criteria presented by Lincken and Guba were used to evaluate the validity and reliability. To analyze the qualitative data, conventional qualitative data analysis and MAXQDA10 software were used. Two themes were obtained in the assessment of interviews and analysis of data: 1) Supportive relief; 2) Therapeutic support.

**Results:**

Some of the participants highlighted the role of social and family support in coping with the disease, and considered social communication and continued support in the form of empathic relationship as a turning point in their lives. The absence of a fertility specialist in the cancer treatment system was the main complaint of most participants. The results showed that receiving support from family and the healthcare system is the most important perceived priorities in breast cancer patients.

**Conclusion:**

The results of this study show the importance of social support as a perceived priority in breast cancer patients to improve their quality of life. Development and reinforcement of the supportive network seem to be essential to provide the best possible help to these patients.

## Introduction

In the world, every three minutes one woman is diagnosed with breast cancer, and a total of one million women with breast cancer are diagnosed annually (Singh and Jangra, 2013). The risk of breast cancer development in the lifetime is 12.5% (one out of eight people), and the risk of death from breast cancer is 3.6% (one case out of twenty-eight) (DeSantis et al., 2014). At present, more than seven million people in the world are afflicted with cancer, and the number of new cases is expected to reach from 10to 15 million people by 2020 (Heydarnejad et al., 2011). Breast cancer is the most common type of cancer in women and the second leading cause of cancer-related death in women after lung cancer (Kruk and Aboul-Enein, 2004).

The prevalence of breast cancer in the Iranian women’s population is 20 new cases per 100,000 women. If the population of Iranian women is estimated at 30 million, there will be 6,000 new cases of breast cancer among them per year (Montazeri et al., 2003). Five-year survival in these patients is 48-84% in different centers (Fouladi et al., 2011). Breast cancer affects the physical, sexual, psychological and social aspects of the afflicted person and her family (Matsuda et al., 2014), so that adaptation to new conditions can lead to tensions in the patient’s personal and family life (Landmark et al., 2008)

Faced with the diagnosis of breast cancer, the patient goes through several psychological stages. The world of a woman with cancer is suddenly and disastrously destroyed, a vague and ambiguous sense of disappointment about the future covers her face and she becomes disappointed, confused and disorganized, but nobody understands her feelings profoundly (Ferlay et al., 2010), while she is heavily in need of support.

Loss of one or both breasts in women with breast cancer causes a feeling of amputation in the affected person (Park et al., 2015). This loss of organ in the affected woman is associated with altered mental image, reduced female sentiments and the sense of attractiveness and sexual attraction, as well as anxiety, depression, impulsiveness, embarrassment, fear of relapse, thinking of rejection, and death thoughts (Al-Azri et al., 2013). These patients need support to be adapted to their new requirements to return to their normal lives (Park et al., 2015).

The results of various studies indicate that after exposure to cancer, the patients mention different priorities for their lives. The need for support from the medical staff (Esmaeili et al., 2012), information (Missel and Birkelund, 2011), acquaintance or support groups (Fu et al., 2008), specialized counseling and relaxation (Esmaeili et al., 2012), the requirement to talk about a new social identity (Esbensen et al., 2008) and more understanding on the part of family (Woodgate, 2005) are among the issues mentioned in various studies.

The need for support as a top priority for cancer patients is a vital and multidimensional requirement that should be continuously met inpatients. The medical staff often considers treatment support as the priority, while the patients give priority to relief support. In spite of the high emphasis on the support for cancer patients, the results of studies show that cancer patients and their families in Iran criticize the lack of a comprehensive and consolidated support network on the part of physicians and nurses, which calls for attention, review and new pieces of trainings in this regard (Kamian et al., 2014).

Researchers have conducted many studies to understand the perceived priorities of cancer patients. From the viewpoint of researchers, social support as an important factor in amelioration of post-traumatic disorder and growth after trauma was one of the most important perceived priorities of women with breast cancer. The results of studies showed that a lower level of social support caused more symptoms of post-traumatic stress disorder in survivors (Morris et al., 2012).

The support by medical staff alongside family support plays an important role in coping with the disease and restoring the functions after cancer (Morris et al., 2012; Sammarco and Konecny, 2008). As a strong and rescue force, support is associated with a successful encounter of individuals with cancer, adaptation to problems with less distress, a greater sense of control and the perception of being understood by others (Lee et al., 2004; Shrestha et al., 2017). In a study on young women with breast cancer, Samarquo (2001) stated that such patients needed more social support. He believed that due to the nature of cancer, young women are more distanced about their plans and aspirations. The fear of disease relapse, sexual (Lee et al., 2004) dysfunction, reproductive disorders and the effect of disease on family along with the impairment of normal course of life are the issues highlighting the need for support in young women with breast cancer (Sammarco, 2001). 

The priority of people in receiving support varies by age. Younger patients, for example, are more concerned about physical appearance, reproductive power and disease relapse, while older patients are more worried about pain, physical symptoms, and family support (Knobf, 2007). Aqabarari et al. In their study showed that social support greatly affects the recovery of patients with breast cancer (Aghabarari et al., 2007). Northouse also identified social support as an important priority for the treatment of cancer in his study (Northouse and Peters-Golden, 1993).

Social support creates a positive self-image leading to the development of hope, love and satisfaction, self-acceptance and reduced anxiety, which increases the person’s adjustment and adaptability to the disease, providing her with an opportunity for self-development and progression (Thoits, 2011). Helgeson in their study identified social support, which was considered as a barrier against the negative outcomes of illness and treatment, as the most important priority of cancer patients (Helgeson, 2003).

Social support increases the person’s ability to adapt to the disease, which helps them have a better feeling of themselves and increases their ability to cope with the conditions (Kroenke et al., 2006). 

Regarding the prevalence of breast cancer among Iranian women and its undesirable effects on the lives of the affected people and their families, it is necessary to reflect and address this issue by identifying the priorities of patients to reduce disease complications. Considering the stress caused by this disease, social support is one of the most important factors increasing the ability to cope with cancer, which improves mental and physical health in the patients. It seems that attempts to reduce stress in patients by identifying their perceived priorities can greatly reduce the burden of breast cancer and improve the quality of life. Regarding the increasing number of cancer patients and the nature of this disease, the need to recognize and understand the priorities and problems of patients after the diagnosis of cancer is of high importance. Therefore, this study was designed and implemented to determine the perceived priorities of women with breast cancer.

## Materials and Methods

This is a qualitative-quantitative successive combined study conducted in 2016 based on content analysis. This type of research depends on establishing a direct relationship with the participants and observing their behavior. 

This study was designed based on the search for perceived healthcare obstacles in women with breast cancer; they had passed the disease phase and were at the recovery stage. These patients expressed their important challenges from the beginning of the disease diagnosis; hence, purposeful sampling was used in this study. In this method, the researcher tried to choose the sample members based on the objectives of the study, which were designed according to information requirements. In targeted sampling, the subjects are recruited because of having specific information on the research topic (Boehmke and Dickerson, 2006). The samples were chosen with the highest variation in terms of age, education level and social class. 

*Inclusion criteria*

The criteria for inclusion in the study were women aged 18-40 years who survived from breast cancer, lack of hormone-dependent breast cancer, patient’s satisfaction for participation in the study and absence of contraceptive indications. 

The semi-structured open interview was used for data collection. The subjects of this study had completed all the treatment steps, and at the time of the study were referred to Motahari Clinic’s Research Center for Breast Diseases affiliated to Shiraz University of Medical Sciences for follow-up.

Before formally starting the personal deep semi-structured interview, the patients were provided with oral explanations on the type of research and its goals during a preliminary introduction session and permitted the researchers to conduct and record the interviews. Adequate explanations were presented on the voluntary basis of participation, the confidentiality of data and the ability to withdraw from the study at any stage. Individual interviews were privately conducted using open questions and lasted 45 minutes to one hour on average. The interviews began with general statements such as “Please tell us the limitations and constraints that have been hurting you after diagnosis of cancer”. Subsequently and gradually, the interview progressed towards deep questions, such as “What was the trend of your constraints during the treatment?”. Now, after completion of treatment according to your physician, is there any obstacle to make you worried?” Exploratory questions were asked based on the information provided by the participants. The researcher explained the questions if needed, and posed inquiries (like how) for better understanding of the participants’ responses during the interview. The interview continued until reaching saturation. In the present study, the data were saturated by interviewing 15 participants.

After conducting the interviews, the tape playback was written down and carefully checked several times to ensure the accuracy of the work. Next, the significant units were identified and summarized using a description that was attempted to have the closest meaning to the text. Then, a list of codes was prepared and placed at a more abstract level through review of similar codes using a reductionist and inductive approach.Afterward, the classes and subclasses were specified through semantic comparison. The data analysis process was based on the steps presented by Granhiem and Landman. The researcher first prepared the manuscripts of interviews and ensured their accuracy through continuous review, and was hence informed of the course of the study. The whole interview, all the paragraphs, sentences, and words were regarded as semantic units. Subsequently, concerning the implicit meaning of the semantic units, their abstraction levels were identified and encoded. The codes received specific labels according to similarity and difference under more abstract classes (Leapet al., 2010).

Dependability, conformability, transferability and credibility methods were used to confirm the accuracy and reliability of the results. Moreover, the credibility of data was increased through interview feedback to participants and asking for review and confirmation of classes derived from the texts of their interviews. The dependency method was used to achieve strength, such that the interviews were submitted to academic faculty members to be inspected (and if necessary revised), and this task was left to the readers to examine the transferability. 

*Statistical analysis*

MAXQDA software (version 11) was used for classification, repeated review, and comparison of different data and citations.

*EthicsCommittee Approval*

Acquiring the necessary permissions and obtaining the code of ethics with the characteristic IR.SBMU.PHNM.1396.903 from the Ethics Committee of Shahid Beheshti University of Medical Sciences for the presence of the researcher and the necessary coordination.

## Results

This research was a qualitative study conducted by a content analysis approach. Two themes were obtained through the assessment of interviews and data analysis: 1) Supportive relief; 2) Therapeutic support. Each of these themes is described with relevant classes below (Table 2).

In this study, 15 women having breast cancer with an average age of 33 years were enrolled. 40% of them (6 persons) had undergraduate education and 46% (7 persons) were employed. 93% of the subjects (14 persons) were married and had one child on average (Table 1).

*First Main Class: Supportive Relief*

This main class consists of three subclasses: 1) Adaptation with the disease 2) Fertility 3) Child rearing, each of which includes several codes. Most participants believed that when they were first informed of their cancer, the question was posed in their minds how to adapt to this problem and its resulting physical changes in family and social life. Some of the participants highlighted the role of social and family support in coping with the disease, and considered social communication and continued support in the form of empathic relationship as a turning point in their lives so that they believed that their emotional relationship with their spouse and children had improved after their disease.

- Participant C: “*The support from my spouse and daughter proved their deepest love for me, and I realized that I was important in their life*” (35 years old, teacher, four years duration of disease).

- Participant J: “*That they did not leave me alone and always accompanied me played a really important role in fighting with my disease*’’ (37 years old, employed, four years duration of disease).

Some other participants considered cancer as a divine trial and called for spirituality as an approach to overcome pain and suffering.

- Participant A: “*It is very difficult to get away with cancer and its stress, and only by getting close to God and asking help from God can we overcome the disease*” (35 years old, housewife, five years duration of disease). Others believed that in addition to other supports, the individual herself should decide to overcome the confusing feelings. “Participant K: “*Stress and anxiety from cancer disorganized me; I did not know what I was doing. The support of people helped me manage my feelings and come back to life*” (44 years old, employed, five years duration of disease).

Fertility was another issue in this class. The majority of participants in this study acknowledged that the only concern of the healthcare system was the treatment of cancer and the system did not address other needs and desires of them. They believed that fertility and childbearing were the basic needs of each woman to create a positive image of her capability and health, which played an important role in improving their quality of life. Most patients complained of marginalizing the issue of fertility and wanted to modify the attitude of the treatment team towards this issue, as the change in attitude leads to the support of health care system for the reproductive health of patients and provision of systematic and safe approaches to achieve this goal.

- Participant O: “*They think survival is the only goal of treatment*” (33 years old, teacher, four years duration of disease). 

Participant J: “*The physician responsible for treatment never cares for fertility, just cures cancer and does a follow-up, We should also be supported and guided for other needs to achieve full health*”(37 years old, employed, four years duration of disease).

- Participant A: “*I have studied; if the physician gives us information promptly, we can do something to maintain our reproductive power before treatment, so that it is not a threat to our health*” (35 years old, housewife, five years duration of disease). The majority of participants acknowledged that if a complete package containing all the required information for the patients and their initial solutions were available, most needs of them could be met with ease and speed.

Participant G: “*We need a lot of information and cannot repeatedly go to the clinic; no one has time to explain to us. If there was a package containing our necessary information, or an integrated center to meet all our needs, it would be extremely helpful*”.

Parenting was another topic in this class. All the participants in our research mentioned parenting as a major concern for which they needed support and help. Patients who did not yet have a child thought that the feeling of health, promotion of hope and self-confidence were concerned with this matter. Those who had a child and experienced a motherly feeling considered parenting both as a concern and an opportunity. The anxiety of death and loneliness of the child were annoying matters that heavily preoccupied the thoughts of some of our participants. Besides this attitude, there was another view that considered parenting and its consequent motherhood love and interest as a reason to try to survive and expressed the support of children highly effective in their health.

- Participant N: “*When I first became aware of my cancer, I was just thinking of the fate of my children after me*“(39 years old, employed, three years duration of disease).

- Participant K: “*I just have to survive for my children. I tried to fight my disease as much as possible to avoid the disappointment of my children*” (44 years old, employed, five years duration of disease).

- Participant I: “*Health is not just the healing of cancer, having a child is a part of my well-being*” (18 years old, student, two years duration of disease).

*Second Main Class: Therapeutic Support*

The absence of a fertility specialist in the cancer treatment system was the main complaint of most participants. Numerous problems arising during treatment and requiring referral and counseling to a fertility specialist led most participants to demand therapeutic support of a fertility specialist for fertility counseling before and after treatment and during remission, care during pregnancy and breastfeeding, advice and treatment of gynecologic disorders from which the patients suffered during treatment, as well as counseling for sexual problems resulting from treatment and its associated stress.

Participant E: “*During our treatment, we encounter many gynecologic problems, from periodic disorders to a reluctance to have sexual intercourse with our spouses. An expert counselor who has completed his education and has time and patience is necessary for these problems*”(37 years old, housewife, five years duration of disease).

Participant J: “*Pregnancy does not matter to anyone in these centers. I think it would be very good if there was a fertility specialist to visit cancer patients to examine and provide guidance because of their special conditions (37 years old, employed, four years duration of disease)*”*.*

- Participant I: “*I have not got married; they did not tell me anything about my prospective pregnancy. Now, they say it may be too late. If a fertility specialist had advised me before starting the treatment, I would have thought of pregnancy first”*. (18 years old, student, two years duration of disease).

Psychological counseling was another issue for which the participants requested support. The stress of cancer diagnosis and treatment caused aggressive states, fatigue, and depression in patients, resulting in mood disorder and conflict with the spouse and child.

Participant O: ”*I have become nervous; I’m bored. When I’m talking to my spouse, it’s as if we were looking for an excuse to fight and get involved together. It was a great help if only they introduced a psychologist for counseling during our treatment” *(33 years old, employed, three years duration of disease).

Participant L: “*Since I have a constant clash with my daughter, she tries to go to her aunt’s or grandmother’s house. When she is at home, she spends time with her father most of the time”* (33 years old, teacher, three years duration of disease).

The need for psychological treatment to adapt to post-disease conditions was another interesting point of the interview.

Participant A: “*Sometimes, we are fed up with our lives and have difficult times. There should be a psychologist in these conditions to save us*” (35 years old, housewife, five years duration of disease).

All the participants of this study complained about the lack of easy and timely access to the information they needed. They acknowledged that they encountered some problems during treatment that required early action, while there was no possibility of continuous referral to the treatment center and full-time access to the physician. There were no full-time physicians. The participants stated the common problems that might afflict most patients, and access to a comprehensive healthcare information database, the development, and availability of books and CDs and provision of information centers with easy personal or on-call access were the best, most logical and easy to use supportive approaches to meet this request from the viewpoint of patients.

- Participant A: “*For example, I’m sick; I want to ask if there is any problem with this drug, I need to go to a specialist, or it is a natural complication. If there is a phone call, I’ll soon find the answer to my question, but now very often I tolerate the pain because of the long-distance and preoccupation. I then suffer from worse complications*”(35 years old, housewife, five years of disease duration).

- Participant I: “*When I come here, we cannot ask questions because of the crowd, but if there is a book, we will look at the book and find the answer*”.

The nature of cancer treatment leads to physical symptoms and changes in the appearance of the patient. Breast cancer is associated with damage to an organ in the body that is the symbol of femininity, causing a worse condition for the patient. The use of prosthetics, wig, and tattoos was another therapeutic care strongly needed by the participants.

Participant M: “*I stay at home because of my appearance. I have an asymmetrical chest, I have lost my hair and eyebrow due to chemotherapy. I do not know in what conditions I can have a prosthesis or whether tattoo harms me. I even cannot go to hairdresser; I do not like to explain; if only they introduced a specific center to us where I could go”* (33 years old, housewife, three years disease duration).

- Participant F: “*Before realizing that I had cancer, I sought treatment for infertility. It is a nightmare for me when I think that cancer and chemotherapy have complicated the problem of infertility. Now, I have realized that I could have done something before starting the treatment, but no one guided me in this respect*” (35-year-old, housewife, three years disease duration).

- Participant O: “*No one talked to me about pregnancy before my treatment; I and my husband only thought of cancer treatment, which has destroyed the fertility power, and we are both frustrated from this fact*” (33 years old, teacher, three years disease duration) (Rostaminejad et al., 2017).

## Discussion

The majority of breast cancer patients experience varying degrees of vulnerability due to the nature of their disease, the supportive therapeutic conditions and community culture after being aware of disease diagnosis (Ashing-Giwa et al., 2004), and have a high level of conflict in their personal, family and social life for adaptation to the disease (Fobair et al., 2006). They need support to adapt to their disrupted needs and conditions to return to their normal lives (Zhanget al., 2010).

In this study, 15 women with breast cancer were interviewed indepth. As noted above, after scrutinizing the results, the priority of the majority of the participants was to have access to a supportive structure to meet their needs and resolve the conflicts. Research suggests that social support has an important role in the adaptation of cancer patients to the disease and its conditions (Bernhardson et al., 2007).

The results of this study showed that the patients sought specialist and therapeutic services at the beginning of diagnosis, but with the progress of treatment and passage of time, support of family members and others as well as receiving proper counseling became more fundamental to them. In our research, the search for support was among the most important strategies of patients in an attempt to adapt to the disease. From the perspective of the participants, support meant the provision of relief and treatment processes, including counseling and information; physical, financial and emotional protection; appreciation and consideration of patients’ needs; and helping to foster self-efficacy and hope. The participants likened receiving support to a sedative that increased their tolerance and ability to cope with the disease although it did not lead to definitive treatment. Inline to our findings, in a study on American black women, those who received less support did not adapt well to the disease (Culver et al., 2004). This result was also consistent with that of the study of Lee et al., (2004).

Social support helps individuals to have a better feeling of themselves and be better capable of coping with their conditions by increasing their adaption and adjustment power to the disease (Boehmke and Dickerson, 2006). Higher adaptation to disease encourages hopeful thinking in people and makes them pay more attention to themselves and thetreatment of their disease, therebytaking the necessary steps to eradicate the disease and its complications more seriously to achieve a higher quality of life.

Reviewing the results of this study and similar studies emphasizes the importance of paying more attention to the role of social support in reducing stress in women with cancer. Finally, it seems that the establishment of a supportive structure and training of social support methods to families and treatment team can reduce the level of damage in these patients and improve their life expectancy.

In our study, the tendency toward spirituality as a link to everlasting divine power promoted the ability to accommodate to the situation and adapt to severe physical and psychological stresses in patients. In this study, the participants had reinforced their distancing and separation from the problem by taking advantage of spiritual adaptation, meaningfulness, purposefulness, hope and rebuilding positive thinking. By relying on individual beliefs, religion helps one to assess negative events in a different way, increase their ability and patience to have a stronger sense of control over the incident and, as a result, enhance their adaptability and consistency (Al-Azri et al., 2009; Danhaueret al., 2009). In a study, Hamid assessed the relationship between the effectiveness of religious-based therapeutics on hopefulness and quality of life in patients with breast cancer. The results of this study indicated that religious-based therapeutics is effective in increasing hope and quality of life of women with breast cancer (Hamid et al., 2012) Karekla and Constantinou (2010) in his study described spiritual beliefs and religious practices as an approach to adapt to the disease in breast cancer patients. 

In general, religious confession helps to control emotional stress by relying on beliefs and religious practices. A sense of belonging to the supreme savior, confidence in the assistance of God and benefiting from spiritual support are all sources of religious affiliation and are helpful in the face of disasters. People create adaptability in various ways. Given the prominent role of religion in the Iranian society, it is suggested that more extensive research studies be conducted on different dimensions of spirituality to find an appropriate way to support and care for patients with breast cancer. The experience of maternal sense and parenting power were other points that the participants described as a milestone for their health.

The request of participants for prioritization of the issue of fertility and childbearing by the treatment team is a significant code expressed by the participants who believed that fertility and child-rearing had become a marginal issue from the viewpoint of the treatment team. The fear of lifelong sterility on the one hand and anxiety of death and negligence of children, on the other hand, were the major concerns that occupied the minds of the majority of participants and caused a level of anxiety similar to the diagnosis of cancer. In this situation, the silence of the treatment team transformed fertility into a big question mark and multiplied the complexity of this problem for patients, which prioritized the need to address this issue and receive therapeutic relief and support in this regard. Several studies have examined fertility and childbearing in women with breast cancer (Partridge et al., 2004; Quinn et al., 2009; Quinn et al., 2007; Rosen, 2005; Zebrack et al., 2004).

Since cancer patients are sometimes forced to repress their feelings about childbearing, they become more and more alien to their being. That the treatment system avoids understanding the new experience of motherhood brings about pessimistic thoughts and the offensive state of tiredness, hopelessness, loneliness, and depression in patients. In contradiction with this state, paying attention to the inner sense of parenting in the guise of guidance and support of the treatment team makes the patient look at life with different new thoughts and views. Counseling and guidance for patients to use the assisted reproduction techniques before the onset of cancer treatment opena window to a new world ahead of the patients. This approach can help the patients to minimize their negative psychosocial effects, maximize their sense of health and minimize the fear of losing female identity. Supporting the patients through timely education, early intervention, presentation of information, explanation of therapies and methods of treatment, as well as guidance to make the right decision in difficult circumstances will greatly help the physical and mental health of the affected people, increasing their life expectancy and strengthening the motivation to begin a new life.

**Table 1:** Demographic Characteristics of the Participants (n=15)

Percent	Number	Number of Children	Percent	Number	Age
13.33	2	does not have	6.66	1	>40
60	9	1	40	6	40-35
26.66	4	2	40 13.33	6 2	35-30 30<
		
Percent	Number	Husbandʹs education	Percent	Number	Education
6.66	1	Less than diploma	13.33	2	Less than diploma
20	3	diploma	26.66	4	diploma
6.66	1	Assistant	13.33	2	Assistant
66.66	10	Bachelor	40 6.66	6 1	Bachelor Master of Science
		
Percent	Number	Husbandʹs Job	Percent	Number	Patient occupation
66.66	10	Clerk	26.66	4	Clerk
33.33	5	Free job	20 53.33	3 8	Free job Housewife
		
Percent	Number	Marital status	Percent	Number	Type of treatment
93.33	14	Married	66.66	10	Mastectomy
6.66	1	Single	33.33	5	Lumpectomy

**Table 2:** Perceived Priorities as Mentioned by Breast Cancer Patients

1/Adapting to changes	Adaptation with the disease	Supportive Relief	Perceived Priorities
2/Coping with illness 3/Religious approach and communication with the creator of the universe 4/Highlighting the family's supporting role 5/ Management of feeling			
1/Developing a service package 2/Changing the negative beliefs of the treatment team 3/Changing the Absolutism of the Physician's Thinking only to Cure the Disease 4/Paying attention to the needs of the patient 5/Inducing the sense of fertility ability in the patient 6/Helping to promote reproductive health 7/Improving the status of information transfer	Fertility		
1/Reducing inefficiency in upbringing the children 2/Eliminating the mother’s concern about her child staying alone 3/Decreasing fear of death 4/Improving hopefulness 5/Increasing self-esteem 6/Enhancing reproductive ability 7/Improving patient health 8/Increasing maternal affection	Child rearing		
Sexual Health and Fertility:	Using expert opinion	Therapeutic Support	
1/Pregnancy counseling before starting treatment 2/Pregnancy counseling during remission 3/Prenatal care counseling 4/Advice on the fetus care during pregnancy 5/ Baby care counseling 6/ Lactation counseling 7/Marital counseling 8/Women's diseases			
The field of psychology:			
1/Mood disorder counseling 2/Behavioral disorders counseling 3/Proper interaction with the spouse 4/Counseling to deal with the child 5/Helping to improve coping ability			
1/Access to comprehensive care information database 2/Compilation and availability of books and educational CDs 3/Providing information centers with easy access to the office or phone	Informing		
1/Use of fertility assisted methods 2/Use of prosthesis 3/Use the wig 4/Use tattoos	Provision of specialized medical services		

This approach can help the patients minimize the negative psychosocial effects of their disease and the fear of losing female identity in themselves, which induces a sense of well-being in patients. Supporting the patients through timely education, early intervention, and presentation of information, explanation of treatment methods and the guidance of the patients to make the right decision in difficult circumstances will greatly help the physical and mental health of the affected people, increasing their life expectancy and strengthening their motivation for a new life.

Cancer patients need help for life-saving adaptation to their disease (Pinquart and Duberstein, 2010). The complications of the disease, suffering of the long-term treatment period and the horror of terrible nature of cancer were the reasons for the need of continuous contact with the treatment team in our participants, which had made easier access to specialists and reception of their support a priority for the patients.

Easy and continuous access to the medical team involved in the treatment can lead to less stress in patients in the face of sudden complications of the disease and treatment, thereby mitigating one of the stressors of their life (Lee et al., 2004; Schroevers et al., 2010). Psychological consultation and other specialized counseling such as fertility and breastfeeding advice increase the sense of self-efficacy in patients and facilitate the confrontation of potential conditions, promoting their ability to adapt to the conditions.

Participants in our study stated easier access to specialists and counselors, receiving medical aids such as prosthetics, access to comprehensive databases such as books and CDs as their supportive therapeutic and palliative priorities. Patients considered such support as an important palliative approach for their concerns and requested paying more attention to perceived priorities to achieve more tranquility. In a study on young women with breast cancer, Sammarco and Konecny (2010) stated the need to various supports in patients in different ages, and that the lack of attention to these requirements is associated with decreased quality of life of patients.

Studies similar to ours also show that therapeutic and relief protection has a positive effect on physical, psychological, social and economic well-being, improving the quality of life of patients, causing a good sense of life better overall assessment of life and more effective confrontation with the disease (Boothroyd and Fisher, 2010; Keating et al., 2011; Lee et al., 2004).

The compilation of a comprehensive database scientifically and understandably can be a desirable strategy to promote the information priority of patients, reduce unnecessary, annoying and frustrating referrals, as well as the stress of confronting sudden complications in patients.

In the present study, a large number of participants attributed the anger and fatigue from the long-term course of diagnosis and treatment as a factor for suffering of themselves and their families, and described their offensive behavior and lack of mutual misunderstanding as a function of this feeling, as well as the breach between the affected person with their spouse and children. Training on coping and behavioral skills in response to the disease and its related problems through raising awareness was another priority of the patients. Taking steps for further involvement of spouses in the treatment course, counseling with the spouses of the affected people and training communication methods, as well as supportive and sedative techniques are among the solution suggested to solvingthis problem. Psychological counseling induces a sense of better control with less distressing experience, improved self-esteem and reduced effects of adverse events on life, which has an impact on health behaviors and treatment outcomes, causing improved mental relaxation and family correlation in patients.

It seems that the treatment staff should provide the positive effect of supportive behaviors in adaptation to the disease by enhancing support groups and raising the awareness of the patients for improved behavior of patients in all dimensions. Therefore, after understanding the patients’ priorities, it is necessary to continuously review their supportive needs and step up the support sources of the healthcare system, taking steps to improve the quality of life of patients with breast cancer with the help of society and participation of family members.

In conclusion, understanding the priorities, needs and beliefs of cancer patients have a potential value in the cancer care system to assess the patients’ cognitive status and develop supportive methods to cope with problems. Patients need to receive social support to promote emotional and physical well-being to adapt to their new conditions of life. The design and implementation of supportive interventions in therapeutic, emotional, social and spiritual fields have significant protective effects on cancer patients.
